# Three-dimensional models increase the interobserver agreement for the treatment of proximal humerus fractures

**DOI:** 10.1186/s13037-020-00258-2

**Published:** 2020-08-06

**Authors:** Luiz Fernando Cocco, André Yui Aihara, Carlos Franciozi, Fernando Baldy dos Reis, Marcus Vinicius Malheiro Luzo

**Affiliations:** 1 Orthopedics Center from Hospital Samaritano, Americas Medical Service, São Paulo, Brazil; 2 Department of Orthopedics and Traumatology, (DOT/UNIFESP) - Escola Paulista de Medicina, Universidade Federal de São Paulo, Rua Napoleão de Barros, 715, 1o andar, São Paulo, SP CEP 04024-002 Brazil; 3 Department of Diagnostic Imaging, UNIFESP, São Paulo, Brazil

**Keywords:** Three-dimensional models, 3D-models, Augmented reality, Holography, Proximal humerus fractures

## Abstract

**Background:**

The agreement for the treatment of proximal humerus fractures is low. Interpretation of exams used for diagnosis can be directly associated with this limitation. This study proposes to compare the agreement between experts and residents in orthopedics for treatment indication of proximal humerus fractures, utilizing 3D-models, holography (augmented reality), x-rays, and tomography as diagnostic methods.

**Methods:**

Twenty orthopedists (ten experts in shoulder and elbow surgery and ten experts in traumatology) and thirty resident physicians in orthopedics evaluated nine fractures of the proximal humerus, randomly distributed as x-rays, tomography, 3D-models and holography, using the Neer and AO / OTA Classifications. After, we evaluated the interobserver agreement between treatment options (conservative, osteosynthesis and arthroplasty) and whether the experience of the evaluators interfered with the results.

**Results:**

The interobserver agreement analysis showed the following kappa-values: κ = 0.362 and κ = 0.306 for experts and residents (3D-models); κ = 0.240 and κ = 0.221 (X-ray); κ = 0.233 and κ = 0.123 (Tomography) and κ = 0.321 and κ = 0.160 (Holography), for experts and residents respectively. Moreover, residents and specialists were discordant in the treatment indication using Tomography as a diagnostic method (*p* = 0.003). The same was not seen for the other diagnostic methods (*p* > 0.05).

**Conclusions:**

Three-dimensional models showed, overall, the highest interobserver agreement (experts versus residents in orthopedics) for the choice of treatment of proximal humerus fractures compared to X-ray, Tomography, and Holography. Agreement in the choice of treatment among experts that used Tomography and Holography as diagnostic methods were two times higher compared to residents.

**Trial registration:**

Registered in Brazil Platform under no. CAAE 12273519.7.0000.5505.

## Background

Proximal humerus fractures are common in orthopedic practice and are likely to become more prevalent with increased life expectancy and the association with osteoporosis [1]. Despite being a routine in orthopedic medical practice, understanding different patterns of shoulder fractures, the number associated injuries, the classification, and proposed treatment remains uncertain. The diversity of treatments has been discussed as a relevant subject in studies involving traumatology and shoulder surgery [1–3].

The interpretation of fractures in the proximal humerus and many other fractures depends on complementary diagnostic tests (usually x-ray and/ or tomography) and the correlation with pre-existing classifications. The widespread and well-known classifications are the Charles Neer, in 1970 [4, 5] and the AO/OTA group - Arbeit Gemeinschaft für Osteosynthesefragen [6]. However, several studies demonstrate low agreement for intra and interobserver reproducibility and the correlation between the diagnosis, classification, and therapeutic proposal involving these lesions [2, 3, 7, 8]. These limitations encourage new studies to improve the classifications known or even alternative diagnostic methods. Recently, Raffaele Russo. et al. [9], Fernando Carlos Mothes. et al. [10] and You W. et al. [11] used 3D-models to improve the surgical programming of proximal humerus fractures and reported good results. Awan OA. et al. [12], using the same three-dimensional models to present acetabular fractures to resident physicians, reported an improvement in the understanding of the particularities of this fracture. In a recent publication from our group [13], we suggested a relevant improvement in the diagnostic agreement among specialists and residents in orthopedics utilizing 3D-models for proximal humerus fractures compared to x-rays and tomographies. In addition, we presented the use of augmented reality (holography) as a diagnostic method to find a way to reproduce the characteristics of these fractures reliably.

In this work, we evaluate the interobserver agreement among four diagnostic methods (x-rays, tomographies, 3D-models, and holography) chosen as the best treatment strategy for proximal humerus fractures.

## Methods

This study was observational, cross-sectional, involving the presentation of proximal humerus fractures as digital x-rays, tomography, 3D-models, and augmented reality to 2 groups of doctors (1 and 2). The images were presented at random, and each group was submitted to four exams. The group was unable to discriminate among the exams during the evaluations.

### Sample size determination

A sample size of 9 cases was determined by statistical analysis, to obtain a 95% confidence interval, with an amplitude of 0.40 for a kappa concordance coefficient estimated at 0.70. A standard deviation of 0.30 was assumed for calculations [14–16].

### Experimental groups

The groups were identified at the time of evaluation as follows:

- Group 1: Twenty experts in shoulder or traumatology from the Brazilian Society of Shoulder and Elbow Surgery (SBCOC) and Brazilian Society of Orthopedic Trauma (SBTO), respectively;
- Group 2: Thirty resident physicians in orthopedics and traumatology from the Department of Orthopedics and Traumatology, UNIFESP / EPM, attending the first, second, or third year of the course.

Likewise, the observers were not identified and were not exposed during the study period.

The x-ray and tomography images of proximal humerus fractures originated from the Hospital Samaritano de São Paulo, Americas Medical Service database. They were used for the 3D-models and holography reconstruction through a specific software used by BioArchitects Company, which was donated for the study. We used the Objet350 Connex 3 printer, with a speed of 12 mm/ hour, 16 μm layers, compatible with Windows 7 and 8. The pieces were printed in resin (photopolymer), with high resolution and in real size, within an average of two hours and thirty minutes per model. The three-dimensional printing models faithfully reproduced the fractures’ original characteristics, such as the number and displacement between the fragments, bone loss and humeral head involvement.

No patient identification information was used to guarantee confidentiality, so we request an exemption from the informed consent form.

**Fig. 1:**
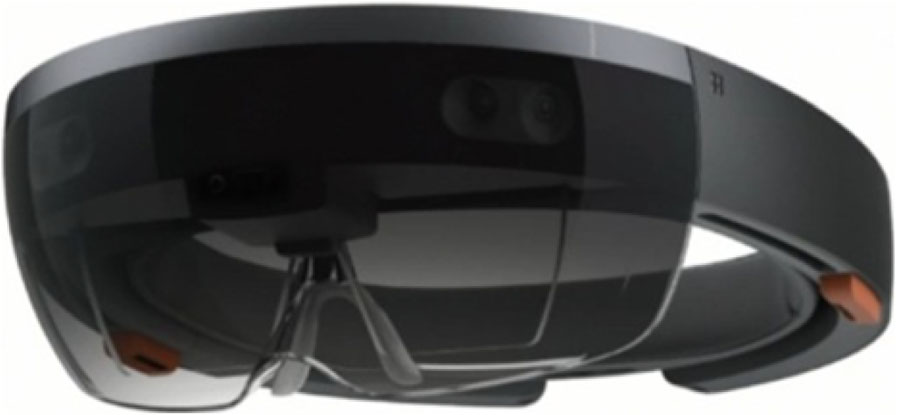
Augmented reality glasses (Microsoft Hololens) to evaluate and indicate the treatment of proximal humerus fractures

To evaluate the proximal humerus fractures through the holographs, glasses were available (Hololens) under the proper positioning of the hologram on the lens according to the user’s viewing angle (Fig. 1).

**Fig. 2:**
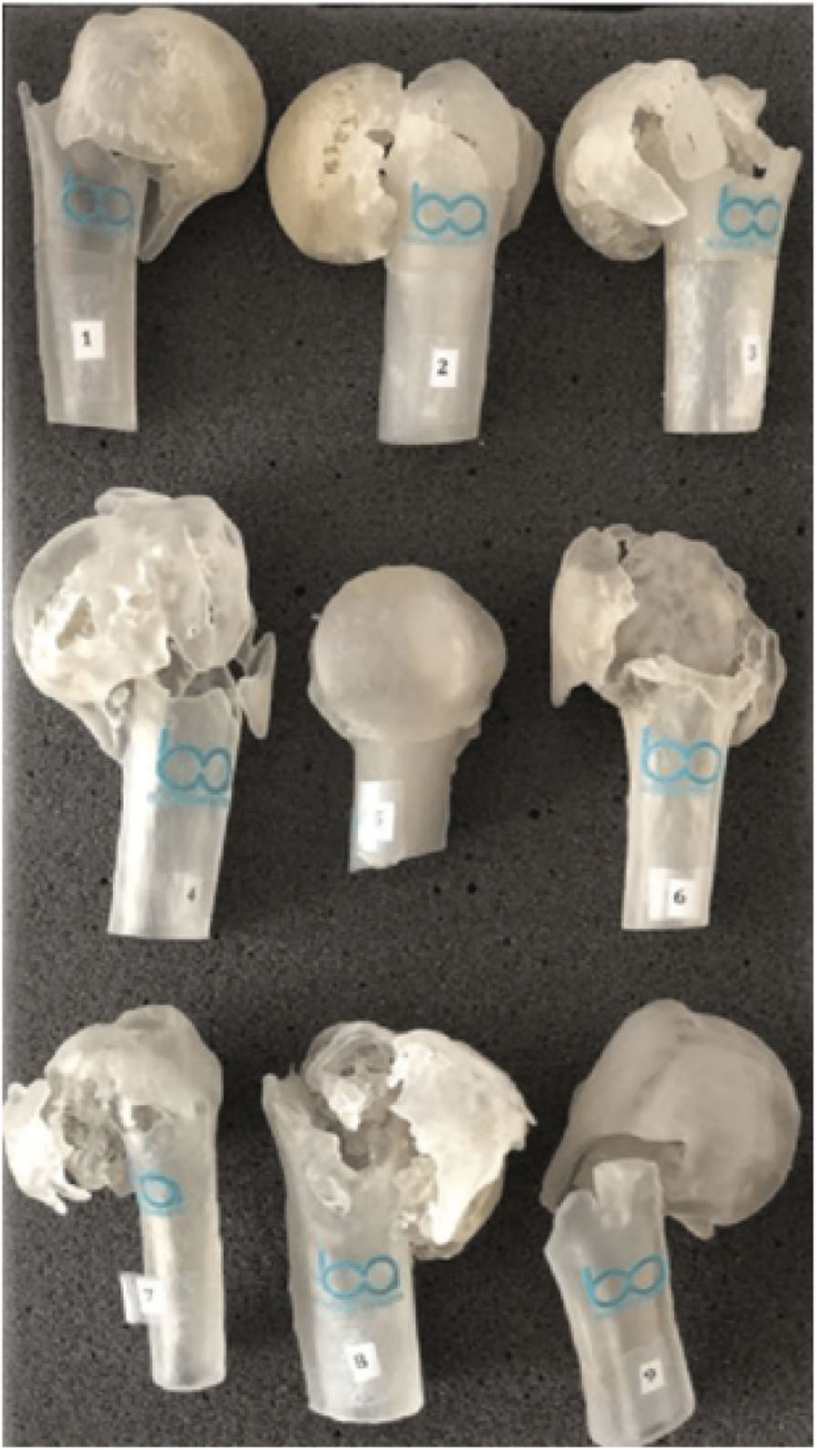
3D-models of proximal humerus fractures used for treatments indication

Biomodels are replicas of patients’ anatomical parts, a three-dimensional model identical to the original. The 3D-models reconstruction, also known as prototyping, is the end product of this process (Fig. 2). Each of the evaluated proximal humerus fractures went through this process, originating the models used for the assessment.

The researchers selected 9 fractures based on the quality of the radiographic images and whether they presented the complete tomographic sequences. Adults (bone growth plate closed) of both sexes were included, without restrictions on laterality. Images with suspected pathological (neoplastic) fractures, infectious diseases, previous fractures in the proximal humerus, congenital deformities, or morphological alterations were not included.

We decided to use only Groups A, B, and C as adopted by the AO/OTA, with correspondence to 2, 3, and 4 parts, respectively, as published in the Journal of Orthopedic Trauma in 2018 [6]. We decided that because there was no objective correspondence of AO/OTA subtypes (A1.1, A1.2, A2.1, A2.2, etc.) and Neer classification.

Therefore, we obtained the following distribution:

1. Three fractures in 02 parts or 11A;
2. Three fractures in 03 parts or 11B or 11C;
3. Three fractures in 04 parts 11C

**Fig. 3:**
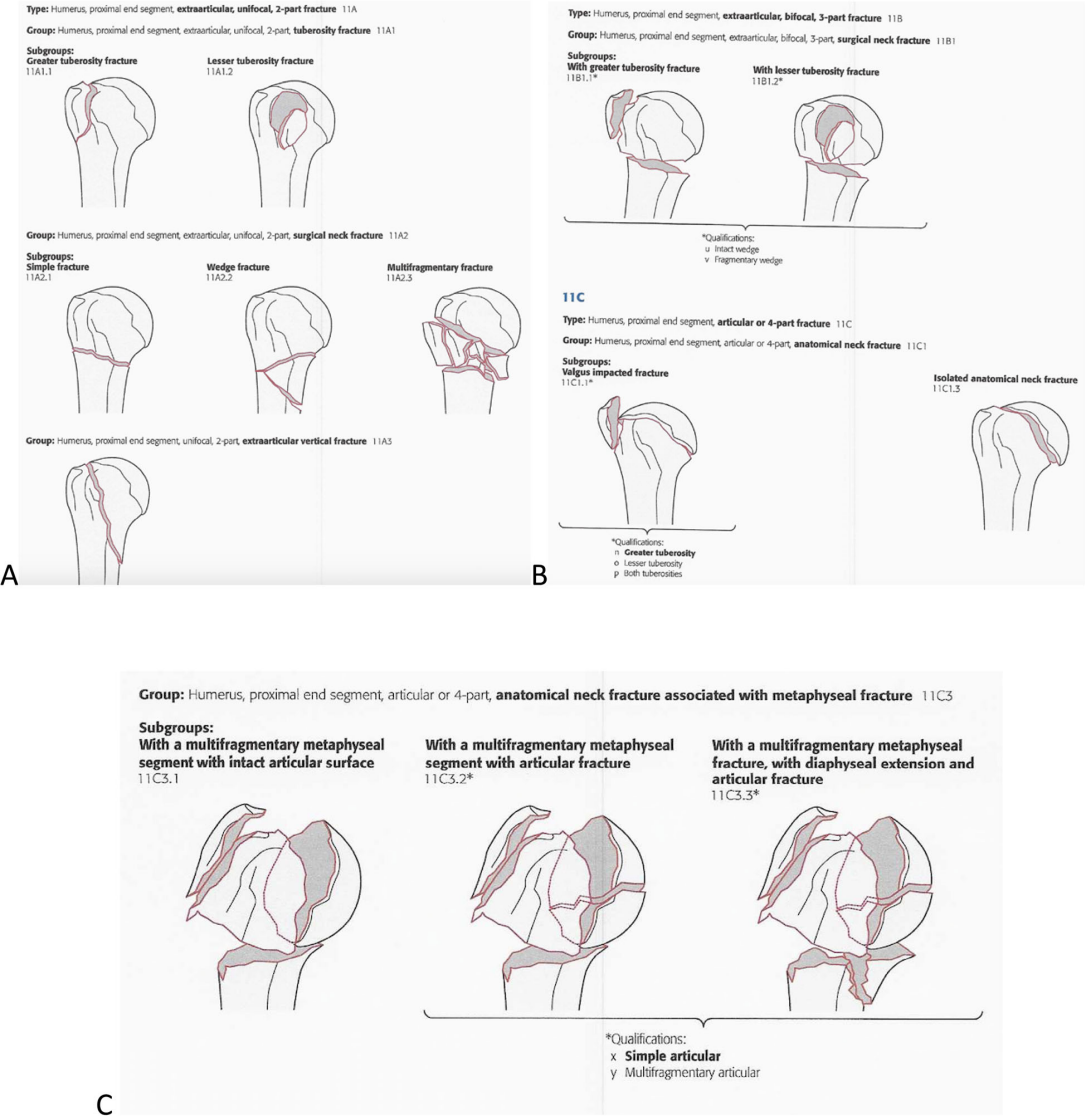
**a**, **b**, **c**: Classification table for proximal humerus fratures; Font: Kellam and Meinberg [6]

During the analysis of the images and the questionnaires filling, the two groups received both classifications in a table, which could be consulted throughout the evaluation, helping the observers choose the answers judged compatible with the exams presented (Figs. 3 a, b, c e 4).

**Fig. 4:**
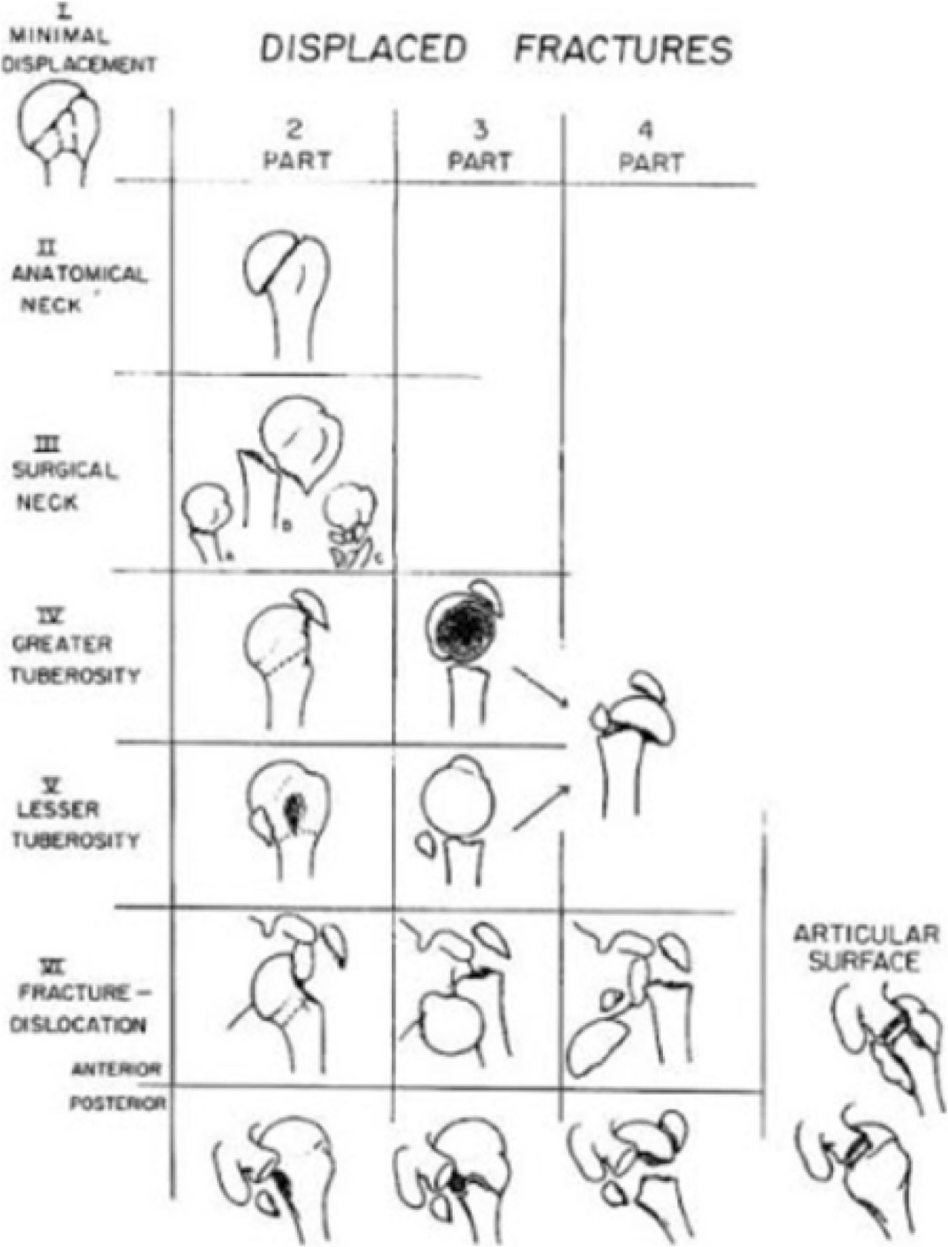
Neer classification. Font: Neer CS (1970) [5]

No clinical or epidemiological information (sex, age, dominance between limbs, associated diseases, fracture period, or mechanism of trauma) was presented to the evaluators. Thus, the indications for fractures treatment were done exclusively by the interpretation of the four diagnostic methods.

**Fig. 5:**
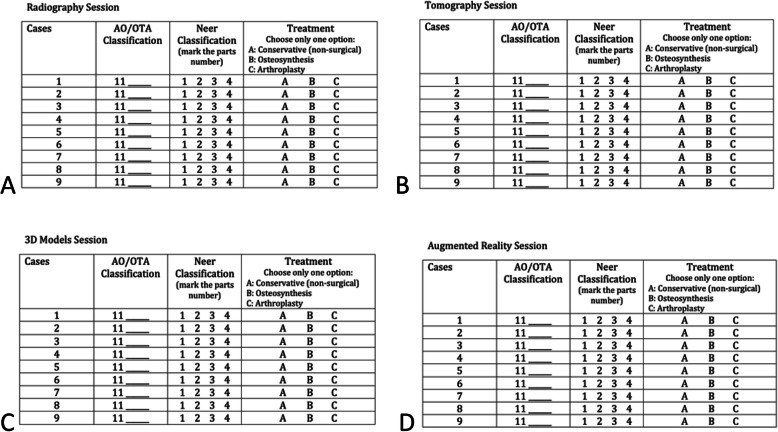
**a**, **b**, **c**, **d**: Questionnaires for classification of proximal humerus fractures and treatment indication using radiography, tomography, 3D-models and holography

The treatment options were presented in a general questionnaire, without specifying non-surgical methods (slings or plaster immobilizations), implants (locked plates, nails, wires, or screws), or prosthesis models (total, partial or reverse). Therefore, the evaluators could decide among only one of the options between treatments: conservative (or non-surgical), osteosynthesis, or arthroplasty (Fig. 5).

### Statistical methods

The global Kappa coefficients [14] were determined to assess the agreement in the choice of treatment among specialists and residents in orthopedics using different diagnostic methods. The statistical analysis was performed in a general and stratified way, using *only the cases* where classifications were in agreement among the observers.

Initially, the association between treatment, type of evaluator and method of diagnosis via Fisher’s exact test was descriptively evaluated, considering independence between the same observer’s response.

The associations between treatments and evaluators (experts or residents) were verified using the Chi-Square test, or alternatively, in case of small samples,^1^ Fisher’s exact test. To verify differences in treatment indication, the standardized adjusted residue was used to identify local differences – cells with absolute values above 1.96 indicate evidence of local associations between the categories.

For all statistical tests, a significance level of 5% was used.

Statistical analyzes were performed using the statistical software SPSS 20.0 and STATA 12.

## Results

Twenty experts in orthopedic trauma/shoulder surgery and thirty residents in orthopedics evaluated nine cases, and the results were tabulated.

**Fig. 6:**
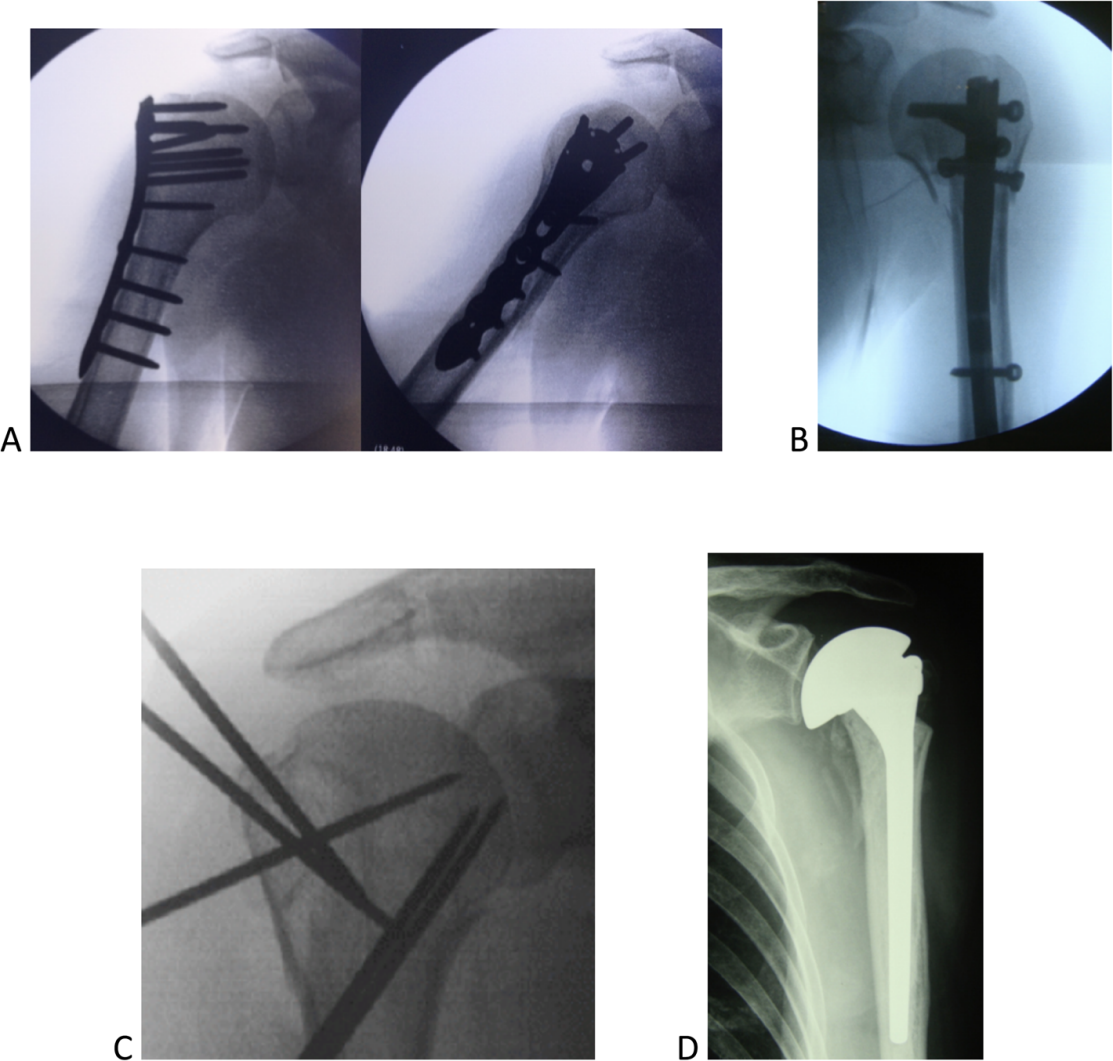
**a**, **b**, **c**, **d** Examples of implants for surgical treatment of proximal humerus fractures. **a**: Plates, **b**: Intramedullary nail, **c**: Steel wires, **d**: Arthroplasty

Table 1 and Fig. 6 show the overall Kappa coefficients by diagnostic methods between experts or residents in orthopedics. For each diagnostic method, agreement and choice of treatment was assessed, dichotomizing each response against the other. The closer the Kappa value is to 1, the higher the agreement. Values ​​close to zero points to an absence of agreement. Landis and Koch [17] provided the rules: A. from 1.00 to 0.81 - Almost perfect agreement; B. from 0.61 to 0.80 – Substantial agreement; C. from 0.41 to 0.60 – Moderate agreement; D. from 0.21 to 0.40 – Weak agreement; E. from 0.0 to 0.20 - Light agreement and F. < 0 – Poor agreement.

**Table 1:** Overall Kappa coefficient for agreement in the choice of treatment for the proximal humerus fractures using four diagnostic methods among experts and residents. *P*-values are shown

	Diagnostic Method							
	X-ray		Tomography		3D-models		Holography	
	Kappa	p	Kappa	p	Kappa	p	Kappa	p
Experts								
Treatment	0.240	< 0.001	0.233	< 0.001	0.362	< 0.001	0.321	< 0.001
Conservative (non-surgical)	0.184	< 0.001	0.185	< 0.001	0.258	< 0.001	0.337	< 0.001
Osteosynthesis	0.182	< 0.001	0.098	< 0.001	0.290	0.001	0.247	< 0.001
Arthroplasty	0.318	< 0.001	0.395	< 0.001	0.471	< 0.001	0.398	< 0.001
Residents								
Treatment	0.221	< 0.001	0.123	< 0.001	0.306	< 0.001	0.160	< 0.001
Conservative (non-surgical)	0.130	< 0.001	0.084	< 0.001	0.223	< 0.001	0.216	< 0.001
Osteosynthesis	0.155	< 0.001	0.045	0.002	0.226	< 0.001	0.099	< 0.001
Arthroplasty	0.330	< 0.001	0.253	< 0.001	0.429	< 0.001	0.197	< 0.001

It was observed that the Kappa coefficients were weak, ranging from 0.123 to 0.362. The agreement in the indication of treatment using 3D-models was higher for experts (κ = 0.362, *p* < 0.001) and residents (κ = 0.306, p < 0.001). For X-ray, the concordances were slightly lower, but similar among experts and residents. Experts showed good agreement using holography compared to residents (two times higher) and very similar to 3D-models. The experts, in general, showed higher agreement in the treatment strategy using different diagnostic methods when compared to residents.

In addition to the agreement analysis between experts and residents, we also performed a comparison of both groups in the choice of treatment using the different diagnostic methods (Table 2). It was possible to verify the differences in the treatment indication (%) between specialists and residents.

**Table 2:** Distribution (%) of treatment indications (non-surgical, osteosynthesis and arthroplasty) for residents and experts using differents diagnostics methods (X-rays, tomography, 3D-models and holography)

	Treatments						Total		p
	Conservative (non-surgical)		Osteosynthesis		Arthroplasty				
	N	%	N	%	N	%	N	%	
Total									
X-ray	42	9,3%	248	55,1%	160	35,6%	450	100,0%	0,451
Residents	29	10,7%	147	54,4%	94	34,8%	270	100,0%	
Experts	13	7,2%	101	56,1%	66	36,7%	180	100,0%	
Tomography	79	17,6%	225	50,0%	146	32,4%	450	100,0%	0,003
Residents	51	18,9%	148	54,8%	71	26,3%	270	100,0%	
Experts	28	15,6%	77	42,8%	75	41,7%	180	100,0%	
3D-models	43	9,6%	250	55,6%	157	34,9%	450	100,0%	0,532
Residents	29	10,7%	150	55,6%	91	33,7%	270	100,0%	
Experts	14	7,8%	100	55,6%	66	36,7%	180	100,0%	
Holography	60	13,3%	262	58,2%	128	28,4%	450	100,0%	0,134
Residents	42	15,6%	158	58,5%	70	25,9%	270	100,0%	
Experts	18	10,0%	104	57,8%	58	32,2%	180	100,0%	

p – descriptive level of the Chi-square test or Fisher’s exact (^a^)

As shown in Table 2, there were differences in the treatment indication using tomography (*p* = 0.003) between residents and experts. It was observed that using tomographic images, experts indicated shoulder arthroplasty more frequently, while residents chose osteosynthesis as the treatment of choice.

## Discussion

The present work evaluated the correlation between different diagnostic methods, the indication of treatment, and the experience of the evaluator. The 3D-models as a diagnostic method showed the highest agreement among interobservers for the treatment of proximal humerus fractures (overall Kappa coefficient), both among experts and residents. Higher concordance based on proximal humerus fractures classification was found using 3D-models in our previous work, when compared to x-ray, tomography or holography [13]. Although 3D-models were the diagnostic method with a higher agreement in the choice of treatment among all interobservers, experts showed overall higher agreement when compared to residents. Therefore, experience time appears to be a significant factor for agreement in the choice of treatment among the four types of diagnostic methods used. However, the results presented here should not be confused with the best treatment for each of the fractures analyzed.

The manipulation of three-dimensional models seems to facilitate the diagnosis and reproducibility using the AO/OTA and Neer Classifications, 1970 [5]. In the case of surgical treatments, preoperative planning can be carried out with 3D-models, allowing the surgeon to train strategies and maneuvers to reposition the deviated bone fragments and choose the implants for each patient before surgery [11, 18, 19]. The choice involves size and models of plates or nails, screws, and others.

Although the prevalence of proximal humerus fractures is relevant and growing worldwide [20], we still have problems with diagnosis and definitions about the best treatment [2, 3, 7, 8, 13, 21–28]. With these findings presented here, we believe that surgeons, while still in training, are influenced by tactile rather than exclusively visual aspects to understand shoulder fractures. The manipulation of 3D-models stimulates areas of reasoning and interpretation that may not be required by merely visual exams such as x-rays, tomographies, and holographies. Similar to the manipulation of 3D models, the palpation of bone fragments is part of the surgical procedure for interpreting the exact fracture pattern. In this respect, probably 3D-models can reproduce this type of stimulus better, justifying the higher agreement obtained in the choice of treatment seen here.

The classifications proposed by Charles Neer, 1970 [5] and the AO / OTA group - Arbeit Gemeinschaft für Osteosynthesefragen [6], the most widespread and used worldwide, were not able to find a relevant reproducibility for diagnosis of proximal humerus fractures [21–23]. It seems logical, therefore, that the best choice to treat patients has uncertainties [2, 3, 13]. Handoll et al. [2] state that there is not enough evidence that surgical treatments are superior to conservative ones. Only a few surgical indications are well established, such as open fractures associated with vascular or neurological injuries that need immediate repair. Because of that, surgeons opt for fixation methods or implants based on their experience and training. Slings, plates, nails, and prostheses are nowadays the therapeutic arsenal used to correlate the patient’s fracture with the surgeon’s skills to decide on the best treatment. Thus, research on topics involving new classifications or diagnostic methods have been presented [6, 13, 18, 20–24, 29, 30] [6, 13, 18, 20–24, 29, 30] and studies with 3D-models are promising [11–13, 19, 31, 32].

Augmented reality or holography is another diagnostic method helping activities that depend on detailed images to show the surgical access routes anatomically. It is composed of tomographic images obtained from fracture analysis and observed by evaluators through special glasses. The beauty and the details of the images do not produce residues, and the innovative and futuristic aspect of the resource encourages the development of new sustainable diagnostic methods by enthusiasts.

The work here assessed the correlation between different diagnostic methods, the indication of treatment, and the experience of the evaluator (experts and residents in orthopedics). We chose not to include clinical or epidemiological information of the nine cases studied (sex, age, dominance between limbs, associated diseases, fracture time or trauma mechanisms). In addition, we decided to keep aside the options between surgical and non-surgical treatments. The alternatives were widely presented as non-surgical or conservative, leaving out the use of slings or plastered immobilizations. The osteosynthesis and arthroplasty methods similarly did not indicate the types of surgical implants (locked plates, nails, wires, screws, models, or types of shoulder prostheses, Fig. 6 A,B,C,D). Therefore, the evaluators were presented with standardized treatment options: non-surgical, osteosynthesis, or shoulder arthroplasty.

Even with significant therapeutic agreement results among experts for all diagnostic methods proposed (as shown in Table 1), we believe that the absence of patients’ clinical variables may have affected the evaluators’ experience and the indications of treatment. However, the inclusion of these parameters would result in a large number of inconclusive variables for the statistical analysis due to the small sample size used here. Thus, the indications for treating fractures of the proximal humerus described here cannot necessarily be reproducible in the presence of a real patient. However, it gives an indication of the diagnostic method and the type of treatment that is more effective and consensual among the observers.

## Conclusions

The agreement for the type of treatment of proximal humerus fractures using three-dimensional models showed, overall, the highest interobserver agreement (experts versus residents in orthopedics) compared to x-rays, tomography, and holography. Moreover, the experts showed two times higher agreement in the treatment that uses tomography and holography, compared to residents.

## Data Availability

All work citation in this work are found in the references section.

## Abbreviations

AO/ASIF: Arbeit Gemeinschaft für Osteosynthesefragen group
CT: Computed tomography
UNIFESP: Universidade Federal de São Paulo

## Footnotes

1 More than 20% of the cells in a contigency table with less than 5 cases.
